# Will PI3K pathway inhibitors be effective as single agents in patients with cancer?

**DOI:** 10.18632/oncotarget.409

**Published:** 2011-12-31

**Authors:** Joan T. Garrett, Anindita Chakrabarty, Carlos L. Arteaga

**Affiliations:** ^1^ Department of Medicine, Vanderbilt-Ingram Cancer Center, Vanderbilt University, Nashville, TN, USA; ^2^ Department of Cancer Biology, Vanderbilt-Ingram Cancer Center, Vanderbilt University, Nashville, TN, USA; ^3^ Breast Cancer Research Program, Vanderbilt-Ingram Cancer Center, Vanderbilt University, Nashville, TN, USA

**Keywords:** cancer, combination therapy, mTOR, PI3K, receptor tyrosine kinases, oncotarget

## Abstract

The phosphatidylinositol 3-kinase (PI3K)/AKT/mammalian target of rapamycin (mTOR) axis regulates essential cellular functions including cell survival, proliferation, metabolism, migration, and angiogenesis. The PI3K pathway is activated in human cancers by mutation, amplification, and deletion of genes encoding components of this pathway. The critical role of PI3K in cancer has led to the development of drugs targeting the effector mechanisms of this signaling network. Recent studies have shown that inhibition at multiple levels of the PI3K pathway results in FOXO-dependent feedback reactivation of several receptor tyrosine kinases (RTKs) which, in turn, limit the sustained inhibition of this pathway and attenuates the action of therapeutic antagonists. This suggests that if used as single agents, PI3K pathway inhibitors may have limited clinical activity. We propose herein that to successfully target the output of the PI3K pathway in cancer cells, combination therapies that hinder these compensatory mechanisms should be used. Thus, combination therapies that target RTKs, PI3K, and mTOR activities may be required to maximize the clinical benefit derived from treatment with these inhibitors.

## INTRODUCTION

The phosphatidylinositol 3-kinase (PI3K) pathway is an important regulator in cell survival, proliferation, and apoptosis. PI3K is a major signaling hub downstream of HER2 and other receptor tyrosine kinases (RTKs). PI3K activates AKT, SGK, PDK1, mTOR, and several other molecules involved in cell cycle progression and survival. The PI3K pathway is one of the most frequently altered networks in cancer [1], with mutation and/or amplification of the genes encoding RTKs such as HER2 (*ERBB2*) and FGFR1, the PI3K catalytic subunits p110α (*PIK3CA*) and p110β (*PIK3CB*), the PI3K regulatory subunit p85α (*PIK3R1*), the PI3K activator mutant K-RAS, the PI3K effectors AKT1, AKT2, PDK1, and loss of the lipid phosphatases PTEN and INPP4B. PI3K is activated by growth factor RTKs and G-protein-coupled receptors (GPCRs). PI3K phosphorylates phosphatidylinositol 4,5-bisphosphate (PIP_2_) to produce the second messenger phosphatidylinositol 3,4,5-trisphosphate (PIP_3_) [2, 3]. Upon formation of PIP_3_, the pleckstrin homology (PH) domain of AKT and PDK1 colocalize at the plasma membrane, resulting in phosphorylation at T308 and activation of AKT. Negative regulation of this pathway is conferred by PTEN and INPP4B, which cleave a phosphate group in PIP_3_ and PIP_2_, respectively. AKT activates the mTOR-containing complex 1 (TORC1) which, via S6K and 4E-BP1, regulates mRNA translation and protein synthesis. mTOR is part of another complex (TORC2), which phosphorlylates AKT at S473 and fully induces its catalytic activity. Little is known about the upstream activators of TORC2 although it is generally thought that growth factors, directly or indirectly, control its activity.

Class IA PI3K isoforms (PIK3Cα, PIK3Cβ, and PIK3Cδ) are heterodimeric proteins that contain a p110 catalytic subunit and a p85 regulatory subunit. Three genes, *PIK3CA*, *PIK3CB*, and *PIK3CD*, encode the homologous p110α, p110β, and p110δ isozymes, respectively. p110α and p110β are ubiquitously expressed whereas p110δ is largely limited to immune and hematopoietic cells. The p110α isozyme is necessary for growth of tumors driven by RTKs, mutant Ras, and/or *PIK3CA* mutations [4]. Conversely, p110β lies downstream of GPCR signaling and ablation of p110β, but not that of p110α, impedes tumorigenesis in PTEN-deficient cells [5]. *PIK3CA* mutations are the most common genetic alterations of this pathway in breast cancer, where ≥80% occur within “hot spots” of exons 9 and 20, corresponding to the helical (E542K and E545K) and kinase (H1047R) domains of p110α. These mutations result in an enzyme with increased catalytic activity through unique mechanisms [6], but both induce similar features of transformation including growth factor- and anchorage-independent growth, and protection from anoikis [7].

The PI3K pathway and its upstream and downstream effectors include many potential targets for drug development in cancer. Drugs inhibiting this pathway at different levels used alone or in combination with chemotherapy, radiation, or other targeted therapies are being evaluated in preclinical and clinical trials and have been summarized recently [8, 9]

## INHIBITION OF THE P13K PATHWAY RESULTS IN FEEDBACK REACTIVATION OF MULTIPLE RTKS

Negative feedback regulation at different levels in the PI3K pathway has been well-documented [10-12]. These feedback loops may have evolved in multicellular organisms to manage growth and nutrient use by individual cells with that of the whole organism [13]. One of the first indications of negative-feedback regulation of the pathway in cancer was found with rapamycin. The macrolide rapamycin and its analogs (rapalogs) complex with FK506-binding protein (FKBP12); this complex then binds to mTOR and, as a result, inhibits the kinase activity of TORC1 but not TORC2. Inhibition of TORC1 and downstream S6K with the rapalog everolimus derepresses levels of insulin receptor substrate (IRS)-1 expression leading to activation of PI3K and phosphorylation of AKT at S473 in both cancer cell lines and tumors of patients [14-16]. These findings may explain the limited clinical activity of TORC1 inhibitors when used as single agents. This observation led to a phase I study of a TORC1 inhibitor and an IGF-IR neutralizing antibody. The combination of both drugs showed interesting clinical activity in patients with luminal B metastatic breast cancer [17]. Inhibition of mTORC1 was also shown to activate the MAPK pathway [18]. In a study of patients treated with the TORC1 inhibitor everolimus, tumors exhibited strong upregulation of ERK phosphorylation. This feedback loop was shown to depend on an S6K-PI3K-Ras pathway.

One approach to circumvent the feedback caused by rapalogs is use of compounds that target the ATP-binding cleft of mTOR and are thus active against both TORC1 and TORC2. Rodrik-Outmezguine *et al*. recently reported that inhibition of TORC1/2 with the small molecule AZD8055 resulted in dephosphorylation of AKT at S473 and a rapid but only transient inhibition of T308 P-AKT. Inhibition of TORC1/2 also relieved feedback inhibition of RTKs resulting in formation of PIP_3_ and rephosphorylation of AKT at T308. Upon recovery of T308 P-AKT, multiple AKT substrates, including FoxO, regained phosphorylation, suggesting T308 P-AKT is catalytically competent. This reinduction of T308 P-AKT and AKT substrates was sensitive to inhibition of AKT but not readdition of mTOR kinase inhibitors, thus demonstrating that such reinduction results from hyperactivity of PI3K. Finally, combined inhibition of mTOR kinase (TORC1/2) and RTKs prevented the rebound of T308 P-AKT and resulted in enhanced cell death and tumor regressions *in vivo* [19].

Similar to the report using TORC1/2 inhibitors, Chandarlapaty and colleagues showed that blockade of AKT (upstream of TORC1 and downstream of TORC2) with an allosteric kinase inhibitor also resulted in enhanced transcription and phosphorylation of multiple RTKs including HER3, IGF-1R, and insulin receptor [20]. These changes are the result of both inhibition of TORC1 and also derepression of FOXO-dependent transcription. Like for rapalogs, inhibition at the level of p110 also results in a compensatory activation of ERK signaling [21]. The activation of HER (ErbB) receptors, as indicated by increased expression of HER3 and binding of adaptor molecules to phosphorylated HER2-HER3 dimers, collectively result in enhanced ERK signaling. The combination of PI3K inhibitors with either HER2 or MEK inhibitors resulted in decreased proliferation, enhanced cell death and superior anti-tumor activity *in vivo* compared with single agent PI3K inhibitors.

## INHIBITION OF P13K IS INCOMPLETE WITH SINIGLE AGENTS

Cancer cells that depend on the HER2 oncogene rely heavily of PI3K activity [22, 23]. In these cells, PI3K is activated by phosphorylated HER2-HER3 dimers. Knockdown of HER3, the adaptor that directly binds PI3K (p85) in these cells, inhibits PI3K and viability of HER2-dependent breast cancer cells [24]. In a clinical trial where patients with HER2+ breast cancer were treated with the HER2 TKI lapatinib, there was significant upregulation of HER3 protein without inhibition of S473-AKT in tumor core biopsies obtained at 2 weeks of treatment [25, 26]. This result was somewhat surprising as tumors were shrinking during therapy with the single agent TKI. The rebound of HER3 protein levels was secondary to the initial inhibition of PI3K-AKT and derepression of FoxO-mediated HER3 mRNA transcription. Recovery of HER3 phosphorylation was secondary to residual HER2 kinase activity and maintenance of ligand-independent HER2-HER3 dimers. Inhibition of HER3 with either siRNA or a neutralizing receptor antibody sensitized to lapatinib, providing evidence that the upregulation of HER3 mRNA and rebound phosphorylation of the HER3 RTK were counteracting the full effect of lapatinib [25].

**Figure 1:**
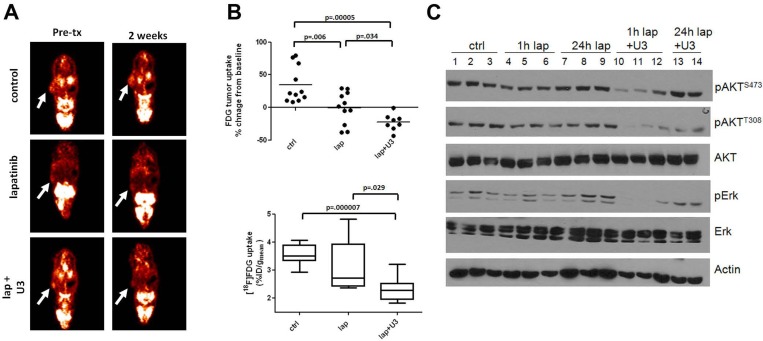
Inhibition of HER3 sensitizes cells to the HER2 inhibitor lapatinib *in vivo* Female athymic mice were injected with BT474 cells as described [25]. Once tumors reached a volume ≥250 mm^3^, mice were randomized to 1) 20 mg/kg normal human IgG i.p. twice a week and vehicle daily via orogastric gavage (control), 2) lapatinib (100 mg/kg daily via orogastric gavage), 3) U3-1287 (20 mg/kg i.p. twice a week), or 4) a combination of lapatinib and U3-1287. Treatment was administered for 4 weeks. (A,B). Tumor-bearing mice were imaged at baseline for [^18^F]FDG uptake, treated as described above and re-imaged on day 14 (*n*=8-11 per group). (A). Images from a representative mouse show [^18^F]FDG uptake pre- and post-treatment. (B). top: Plot of the % change in [^18^F]FDG uptake at day 14 compared to baseline is shown. (B). bottom: Raw values of [^18^F]FDG %ID/g at 14 days post-treatment. Boxes indicate 25^th^ to 75^th^ percentile of values. The solid line indicates the median value and external lines show the complete range. (C). At the end of 4 weeks of treatment, mice were sacrificed either 1 or 24 h after the last dose of lapatinib. Tumor cell lysates were prepared and separated in a 7% SDS gel followed by immunoblot analysis with the indicated antibodies.

The data summarized above suggest that single agent HER2 inhibitors cannot block completely the signaling output from HER2 to HER3-PI3K-AKT. They also suggest that surrogate markers of PI3K activity would be valuable metrics to assess the magnitude of therapeutic pharmacodynamic inhibition in tumors that depend on oncogenes that activate and depend on PI3K. Genes encoding most glycolytic enzymes are under dominant transcriptional control by PI3K/AKT [27]. Further, activation of AKT stimulates glucose import and metabolism [28]. Thus, a rapid decrease in [^18^F]-fluoro-deoxyglucose positron emission tomography (FDG-PET) uptake may represent a surrogate marker of inactivation of the PI3K/AKT pathway. In a recent study, we treated HER2+ BT474 xenografts with the combination of lapatinib and U3-1287, a neutralizing HER3 monoclonal antibody [29, 30]. To determine whether the combined inhibition of HER2 and HER3 with lapatinib and U3-1287 improved the inhibition of PI3K over each single agent, we used FDG-PET imaging. Baseline tumor [^18^F]FDG uptake was measured; mice were then randomized to control vehicle, lapatinib, U3-1287, or both drugs combined and re-imaged on day 14. Treatment with the combination resulted in a statistically significant decrease in [^18^F]FDG tumor uptake compared to both vehicle- and lapatinib-treated mice (Figure 1A and B). Treatment with U3-1287 alone had no effect on [^18^F]FDG uptake (data not shown). At 14 days of therapy there was no statistically difference in tumor volume for any of the treatment groups [25], suggesting that [^18^F]FDG uptake can be used as an early imaging biomarker predictive of response to a PI3K inhibitory therapy. Immunoblot analysis of tumor lysates from tumors harvested at 28 days of treatment revealed greater inhibition of P-AKT and P-Erk in xenografts treated with the combination compared to xenografts treated with lapatinib alone (Figure 1C). After 4 weeks of therapy, tumors treated with the combination exhibited a markedly reduced volume compared to the lapatinib and control arms [25]. These results suggest that a single-agent inhibitor is ineffective at completely blocking the PI3K pathway and that combination approaches are needed to block feedback-regulated pathways thus completing blockade of the PI3K axis.

In a second example, we reported that treatment with the pan-PI3K inhibitor XL-147 (Exelixis) was followed by up-regulation of expression and phosphorylation of several RTKs, including HER3, InsR, IGF-1R, FGFR2/3 [31]. Knockdown of FoxO1 and FoxO3a transcription factors suppressed the induction of HER3, InsR, IGF-1R, and FGFR2 mRNAs upon inhibition of PI3K. In HER2+ cells, HER3 was the main RTK that became reactivated upon treatment with XL-147. Further, addition of lapatinib or the HER2 antibody trastuzumab to XL-147 in mice with established BT474 xenografts resulted in better growth inhibition as well as reduction of tumor levels of P-HER3, P-AKT, and P-S6.

**Figure 2:**
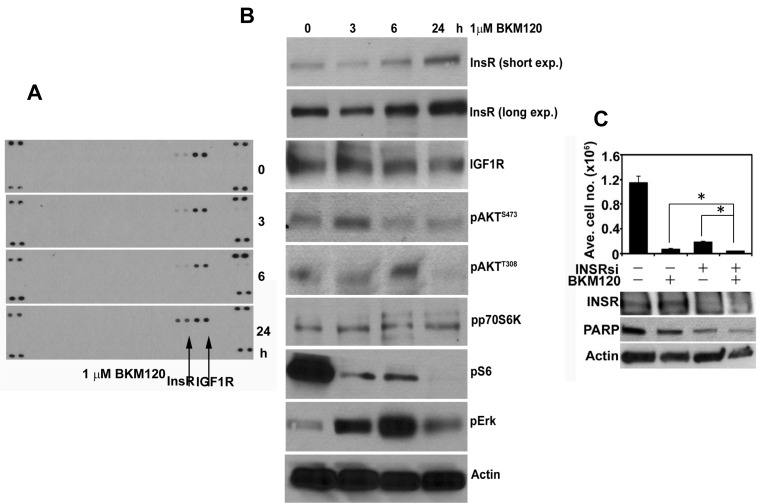
PI3K inhibition in MCF7 cells results in compensatory upregulation of InsR (A). MCF7 cells were treated with 1 μM BKM120 for the indicated time points; at these times cells were harvested and cell lysates prepared. Cell lysates (500 μg) were hybridized with phospho-RTK arrays (R&D Systems) according to the manufacturer's instructions. (B). Fifty μg/lane of cell lysate from A were separated by SDS-PAGE followed by transfer to nitrocellulose and immunoblot analysis with the indicated antibodies. (C). MCF7 cells were transfected with 50 nM of control or InsR siRNA and seeded in 6-well plates followed by treatment with 1 μM BKM120. Seven days after transfection, the cells were harvested and counted in a Coulter counter (top panel). Three days post-transfection (or 24 h after treatment with BKM120) cell lysates were prepared and subjected to immunoblot analyses for InsR, PARP, and Actin (control; bottom panel). Each bar represents mean ± SE cell number in triplicate wells (*p<0.05).

T the biologically relevant RTKs that compensate for inhibition of PI3K-AKT in cancers expressing low levels of HER2 are less clear. We have examined this question in ER+/HER2-negative MCF7 human breast cancer cells which also harbor an activating E545K mutation in *PIK3CA* (p110α) [32]. In these cells, treatment with the pan-PI3K inhibitor BKM120 [33] over a time-course was followed by upregulation of total and phosphorylated InsR at 24 h (Figure 2A,B). The compensatory upregulation of total and P-InsR upon inhibition of PI3K suggested that combined inhibition of InsR and PI3K would synergistically inhibit tumor cell viability. Therefore, we transfected MCF7 cells with InsR siRNA oligonucleotides in combination with BKM120. The combination of InsR knockdown and BKM120 resulted in a statistical reduction in cell growth compared to either treatment alone. This was further confirmed by immunoblot of PARP, a biomarker of cell death (Figure 2C). These data further suggest that upon inhibition of PI3K, PI3K-dependent cells upregulate RTKs that hamper the efficacy of a single-agent PI3K pathway inhibitor. Taken together, the findings presented here and elsewhere [14, 18-21, 25, 31] reveal a complex network of feedback regulation between PI3K, mTOR, and RTKs.

## CLINICAL IMPLICATIONS

These findings have obvious translational implications for the treatment of PI3K-pathway dependent cancers. The relief of negative feedback loops upon inhibition of different levels in the PI3K-AKT pathway and subsequent reactivation of RTKs suggest that, if used as single agents, PI3K inhibitors will have suboptimal clinical activity. It is possible that relief of these feedback loops is also occurring in non-tumor host tissues and thus ameliorating drug-related toxicities. Combined targeting of different levels of the PI3K pathway, i.e., TORC1/2 and p110, RTK and p110; RTK and TORC1/2, etc., in order to more completely suppress its signaling output, have not been fully explored. Whether those combined approaches limit feedback reactivation and, as a result, block the pathway more strongly and/or whether they are clinically well tolerated and feasible remains to be determined. Compensatory feedback may not be limited to RTKs. For example, inhibition of PI3K in prostate cancer cells activates signaling by the androgen receptor (AR) and inhibition of the AR, in turn, activates AKT signaling by reducing levels of the AKT phosphatase PHLPP [34]. Whether similar coordinate regulation of steroid receptors exists in other hormone-dependent tumors remains to be established. Interestingly, however, experimental and clinical evidence suggests that hyperactivation of the PI3K pathway promotes antiestrogen resistance in breast cancer [35], suggesting that simultaneous targeting of PI3K and ER pathways may be effective in ER+ breast cancer [36]. The ability of these combinations to perform in the clinic is currently the focus of active investigation. Ideally, these studies should confirm whether the feedback reactivation seen in the laboratory in cancer cells following PI3K-AKT inhibition is of the same nature in primary tumors.

## INHIBITION OF P13K IS INCOMPLETE WITH SINGLE AGENTS

Cancer cells that depend on the HER2 oncogene rely heavily of PI3K activity [22, 23]. In these cells, PI3K is activated by phosphorylated HER2-HER3 dimers. Knockdown of HER3, the adaptor that directly binds PI3K (p85) in these cells, inhibits PI3K and viability of HER2-dependent breast cancer cells [24]. In a clinical trial where patients with HER2+ breast cancer were treated with the HER2 TKI lapatinib, there was significant upregulation of HER3 protein without inhibition of S473-AKT in tumor core biopsies obtained at 2 weeks of treatment [25, 26]. This result was somewhat surprising as tumors were shrinking during therapy with the single agent TKI. The rebound of HER3 protein levels was secondary to the initial inhibition of PI3K-AKT and derepression of FoxO-mediated HER3 mRNA transcription. Recovery of HER3 phosphorylation was secondary to residual HER2 kinase activity and maintenance of ligand-independent HER2-HER3 dimers. Inhibition of HER3 with either siRNA or a neutralizing receptor antibody sensitized to lapatinib, providing evidence that the upregulation of HER3 mRNA and rebound phosphorylation of the HER3 RTK were counteracting the full effect of lapatinib [25].

The data summarized above suggest that single agent HER2 inhibitors cannot block completely the signaling output from HER2 to HER3-PI3K-AKT. They also suggest that surrogate markers of PI3K activity would be valuable metrics to assess the magnitude of therapeutic pharmacodynamic inhibition in tumors that depend on oncogenes that activate and depend on PI3K. Genes encoding most glycolytic enzymes are under dominant transcriptional control by PI3K/AKT [27]. Further, activation of AKT stimulates glucose import and metabolism [28]. Thus, a rapid decrease in [^18^F]-fluoro-deoxyglucose positron emission tomography (FDG-PET) uptake may represent a surrogate marker of inactivation of the PI3K/AKT pathway. In a recent study, we treated HER2+ BT474 xenografts with the combination of lapatinib and U3-1287, a neutralizing HER3 monoclonal antibody [29, 30]. To determine whether the combined inhibition of HER2 and HER3 with lapatinib and U3-1287 improved the inhibition of PI3K over each single agent, we used FDG-PET imaging. Baseline tumor [^18^F]FDG uptake was measured; mice were then randomized to control vehicle, lapatinib, U3-1287, or both drugs combined and re-imaged on day 14. Treatment with the combination resulted in a statistically significant decrease in [^18^F]FDG tumor uptake compared to both vehicle- and lapatinib-treated mice (Figure 1A and B). Treatment with U3-1287 alone had no effect on [^18^F]FDG uptake (data not shown). At 14 days of therapy there was no statistically difference in tumor volume for any of the treatment groups [25], suggesting that [^18^F]FDG uptake can be used as an early imaging biomarker predictive of response to a PI3K inhibitory therapy. Immunoblot analysis of tumor lysates from tumors harvested at 28 days of treatment revealed greater inhibition of P-AKT and P-Erk in xenografts treated with the combination compared to xenografts treated with lapatinib alone (Figure 1C). After 4 weeks of therapy, tumors treated with the combination exhibited a markedly reduced volume compared to the lapatinib and control arms [25]. These results suggest that a single-agent inhibitor is ineffective at completely blocking the PI3K pathway and that combination approaches are needed to block feedback-regulated pathways thus completing blockade of the PI3K axis.

In a second example, we reported that treatment with the pan-PI3K inhibitor XL-147 (Exelixis) was followed by up-regulation of expression and phosphorylation of several RTKs, including HER3, InsR, IGF-1R, FGFR2/3 [31]. Knockdown of FoxO1 and FoxO3a transcription factors suppressed the induction of HER3, InsR, IGF-1R, and FGFR2 mRNAs upon inhibition of PI3K. In HER2+ cells, HER3 was the main RTK that became reactivated upon treatment with XL-147. Further, addition of lapatinib or the HER2 antibody trastuzumab to XL-147 in mice with established BT474 xenografts resulted in better growth inhibition as well as reduction of tumor levels of P-HER3, P-AKT, and P-S6.

T the biologically relevant RTKs that compensate for inhibition of PI3K-AKT in cancers expressing low levels of HER2 are less clear. We have examined this question in ER+/HER2-negative MCF7 human breast cancer cells which also harbor an activating E545K mutation in *PIK3CA* (p110α) [32]. In these cells, treatment with the pan-PI3K inhibitor BKM120 [33] over a time-course was followed by upregulation of total and phosphorylated InsR at 24 h (Figure 2A,B). The compensatory upregulation of total and P-InsR upon inhibition of PI3K suggested that combined inhibition of InsR and PI3K would synergistically inhibit tumor cell viability. Therefore, we transfected MCF7 cells with InsR siRNA oligonucleotides in combination with BKM120. The combination of InsR knockdown and BKM120 resulted in a statistical reduction in cell growth compared to either treatment alone. This was further confirmed by immunoblot of PARP, a biomarker of cell death (Figure 2C). These data further suggest that upon inhibition of PI3K, PI3K-dependent cells upregulate RTKs that hamper the efficacy of a single-agent PI3K pathway inhibitor. Taken together, the findings presented here and elsewhere [14, 18 -21, 25, 31] reveal a complex network of feedback regulation between PI3K, mTOR, and RTKs.

## CLINICAL IMPLICATIONS

These findings have obvious translational implications for the treatment of PI3K-pathway dependent cancers. The relief of negative feedback loops upon inhibition of different levels in the PI3K-AKT pathway and subsequent reactivation of RTKs suggest that, if used as single agents, PI3K inhibitors will have suboptimal clinical activity. It is possible that relief of these feedback loops is also occurring in non-tumor host tissues and thus ameliorating drug-related toxicities. Combined targeting of different levels of the PI3K pathway, i.e., TORC1/2 and p110, RTK and p110; RTK and TORC1/2, etc., in order to more completely suppress its signaling output, have not been fully explored. Whether those combined approaches limit feedback reactivation and, as a result, block the pathway more strongly and/or whether they are clinically well tolerated and feasible remains to be determined. Compensatory feedback may not be limited to RTKs. For example, inhibition of PI3K in prostate cancer cells activates signaling by the androgen receptor (AR) and inhibition of the AR, in turn, activates AKT signaling by reducing levels of the AKT phosphatase PHLPP [34]. Whether similar coordinate regulation of steroid receptors exists in other hormone-dependent tumors remains to be established. Interestingly, however, experimental and clinical evidence suggests that hyperactivation of the PI3K pathway promotes antiestrogen resistance in breast cancer [35], suggesting that simultaneous targeting of PI3K and ER pathways may be effective in ER+ breast cancer [36]. The ability of these combinations to perform in the clinic is currently the focus of active investigation. Ideally, these studies should confirm whether the feedback reactivation seen in the laboratory in cancer cells following PI3K-AKT inhibition is of the same nature in primary tumors.
