# Erratum: Will PI3K pathway inhibitors be effective as single agents in patients with cancer?

**DOI:** 10.18632/oncotarget.26017

**Published:** 2018-08-17

**Authors:** Joan T. Garrett, Anindita Chakrabarty, Carlos L. Arteaga

**Affiliations:** ^1^ Department of Medicine, Vanderbilt-Ingram Cancer Center, Vanderbilt University, Nashville, TN, USA; ^2^ Department of Cancer Biology, Vanderbilt-Ingram Cancer Center, Vanderbilt University, Nashville, TN, USA; ^3^ Breast Cancer Research Program, Vanderbilt-Ingram Cancer Center, Vanderbilt University, Nashville, TN, USA

**This article has been corrected:** During production, the text of the subsections ‘INHIBITION OF P13K IS INCOMPLETE WITH SINGLE AGENTS’ and ‘CLINICAL IMPLICATIONS’ was duplicated within the article. The corrected version now has the duplicated text removed while preserving the original pagination.

Original article: Oncotarget. 2011; 2:1314-1321. https://doi.org/10.18632/oncotarget.409

